# Effects of a Copper-Deficient Diet on the Biochemistry, Neural Morphology and Behavior of Aged Mice

**DOI:** 10.1371/journal.pone.0047063

**Published:** 2012-10-10

**Authors:** Silvia Bolognin, Federica Pasqualetto, Carla Mucignat-Caretta, Janez Scancar, Radmila Milacic, Pamela Zambenedetti, Bruno Cozzi, Paolo Zatta

**Affiliations:** 1 CNR-Institute for Biomedical Technologies, Metalloproteins Unit, Department of Biology, University of Padova, Padova, Italy; 2 Department of Human Anatomy and Physiology, University of Padova, Padova, Italy; 3 Department of Environmental Sciences, Jozef Stefan Institute, Ljubljana, Slovenia; 4 Division of Pathology, General Hospital, Dolo (VE), Italy; 5 Department of Experimental Veterinary Science, University of Padova, Legnaro (PD), Italy; University G. D’Annunzio, Italy

## Abstract

Copper dyshomeostasis has been suggested as an aetiological risk factor for some neurodegenerative diseases, such as Alzheimer’s disease. However, the precise mechanism at the base of this involvement is still obscure. In this work, we show the effects of a copper-deficient diet in aged CD1 mice and the influence of such a diet on: a) the concentration of various metal ions (aluminium, copper, iron, calcium, zinc) in the main organs and in different brain areas; b) the alteration of metallothioneins I-II and tyrosine hydroxylase immunopositivity in the brain; c) behavioural tests (open field, pole, predatory aggression, and habituation/dishabituation smell tests). Our data suggested that the copper-deficiency was able to produce a sort of “domino effect” which altered the concentration of the other tested metal ions in the main organs as well as in the brain, without, however, significantly affecting the animal behaviour.

## Introduction

Solid widely recognized evidence suggests that altered metal ion homeostasis may be implicated in the aetiology of several neurodegenerative disorders, particularly Alzheimer’s disease (AD) [1], [2], [3] While metal alteration has been clearly established as the primary cause of the disease for few and rare neurodegenerative disorders (*e.g.* Wilson’s disease), its involvement is still a controversial issue for the remaining common disorders. The brain tightly regulates metals trafficking as an important part of its normal activity, and disruption of this delicate equilibrium may have detrimental effects for the whole brain functioning. According to some studies, an increase in brain copper (Cu) concentration could be a risk co-factor for the progression of AD [4]. Cu is an essential trace metal, acting as an important catalytic cofactor in several redox reactions involved in growth and development [5]. However, several studies suggested that it might interact with β-amyloid 1–42 (Aβ_1–42_), and catalyse hydrogen peroxide (H_2_O_2_) generation, using a variety of biological reducing agents as substrates [6], [7]. In the absence of sufficient levels of detoxifying enzymes, including catalase, superoxide dismutase and glutathione peroxidase, H_2_O_2_ can further react with Cu to generate highly toxic hydroxyl radicals *via* the Fenton reaction, becoming a potential neuropathogenic factor. Interestingly, Aβ and amyloid precursor protein (APP) are both metalloproteins that strongly bind Cu.

According to several findings, Cu concentration was lower in the central nervous system (CNS) of AD patients in comparison to age match controls [8], [9] although it increased focally in the senile plaques [10]. AD, in addition, seems to be characterized by an excess of Cu in the brain extracellular space [11] and by an intracellular decrease with respect to the age-matched controls [8]. Nevertheless, pure analytical data are highly controversial, and evidence for a Cu-AD connection is mainly indirect. Cells over-expressing APP exposed to high Cu levels decreased the rate of Aβ secretion [12], an effect replicated in AD animal models by elevating Cu levels in the brain both by genetic [13] as well as by dietary supplementation [14]. Moreover, dietary Cu supplementation, in a transgenic mouse model for AD, increased bioavailable Cu levels in the brain and lowered Aβ levels, suggesting that impaired Cu homeostasis could be linked to the pathology [14]. In fact, increased intracellular Cu availability inhibited the accumulation of Aβ oligomers and protein tau phosphorylation [15]. Nevertheless, a prospective double-blind study in patients with mild AD failed to demonstrate that a 8 mg Cu daily supplementation produced an effect on tau and phospho-tau levels in cerebrospinal fluid (CSF) that represent relevant markers for AD [16]. Diversely, during the 12 months of treatment, Aβ_1–42_ markedly decreased in the control group compared to the Cu supplemented group. However, none of the above studies have demonstrated the existence of a Cu deficiency condition in AD patients [17]. Anyway, other set of studies reported detrimental effects upon dietary Cu: the addition of Cu (0.12 ppm) to drinking water worsened Aβ accumulation and learning deficit in a cholesterol-fed rabbit model of AD, suggesting that Cu may negatively influence Aβ clearance from the brain [18]. Epidemiological studies hinted that high Cu dietary intake, associated with a high saturated and trans fat diet, accelerated cognitive decline [19]. In addition, alteration of APP processing, enhanced Aβ production and tau phosphorylation were detected in young triple transgenic (3xTg-AD) mice after chronic Cu exposure [20].

The metal dishomeostasis, often observed in association with AD, does not correspond in most cases to a dramatic and generalised metal imbalance in the brain, but rather to focal alteration of a certain metal in few specific and critical brain areas or even cellular compartments (for a review see [21]). Consequently, neither the total metal burden of the brain nor the relative concentration of the main biometals is greatly perturbed in several instances.

This complex scenario prompted us to investigate the effects of a chronic Cu deficient diet in aged mice and to assess the relationship with other metal ions in the brain and other selected organs. We chose this experimental model as we wanted to unravel whether a deficiency of an essential metal in aged mice was able to produce a detectable effect in terms of behavioural and biochemical alterations. It has been indeed demonstrated that the dietary intake of essential micronutrients is usually inadequate in elderly [22]. This gap is worsened by loss of appetite, lack of teeth, reduced mineral intestinal absorption and decreased requirement of energy. We also tested whether metal ions act independently or may be linked to promote a sort of domino effect in which changes in the concentration of one element may alter the others, with a consequent variation in oxidative stress markers and animal behaviour.

## Materials and Methods

### Animal Care and Diet

**Figure 1:**
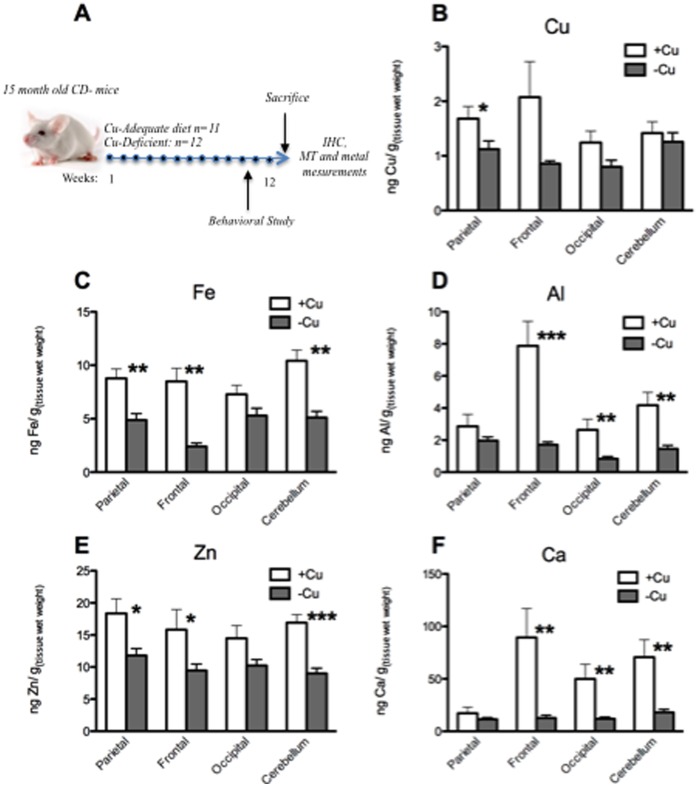
Metal content in the brain. (A) Schematic representation of the outline of the study. 15 month old CD1 mice were fed with a Cu-adequate (CuA) or Cu-deficient (CuD) diet for 12 weeks. Behavioural tests were performed the last 2 weeks of the treatment. Mice were then sacrificed and metal determination, IHC as well as biochemical investigations were performed. (B) Cu, (C) Fe, (D) Al, (E) Zn, and (F) Ca content (µg/g wet tissue) in the parietal, frontal, occipital cortex, and cerebellum. *p<0.05; **p<0.01; ***p<0.001.

Twenty-three male CD-1 mice (15 month old) were purchased from Harlan (Udine, Italy). They were housed one mouse *per* cage under standard laboratory conditions (23±1°C, 55±5% humidity, 12 h light/dark cycle) and had access to bi-distilled water and food *ad libitum*. They were allowed to acclimatize for 1 month before starting the treatment. Mice were randomly divided in two groups for the Cu-deficient (CuD) diet (*n* = 12) and Cu-adequate diet (*n* = 11) and treated for 12 weeks (Fig. 1A). Animals were sacrificed after the 12 weeks of treatment by decapitation. The diet with (6 mg of cupric carbonate *per* kilo) and without Cu was purchased from Mucedola (Settimo Milanese, Italy). Controls were run to check the Cu concentration of the diet upon arrival. All mice were treated in accordance with the policy of the Italian Ministry of Health and European Community laws on the use of animals in research (116-92, art. 5). The procedures used were approved by the Institute of Vallisneri, University of Padova.

### Tissue Sampling

The brains were divided into two halves, one of which was frozen immediately after the decapitation for biochemical measurements and the other one used for immunohistochemistry (see below for details). Other organs were removed, weighted and immediately frozen until biochemical determination of metal ion and metallothionein (MT) concentrations.

### Metal Analyses

Fresh tissues were mineralized in HNO_3_ Suprapur (Merk, Milan, Italy) at 70°C for 24 h. After digestion, the solution was brought to the final volume using milliQ water and filtered with 0.2 µm pore size (Sigma, Milan, Italy).

The concentration of Cu, iron (Fe), calcium (Ca), and zinc (Zn) in tissues was determined by a Perkin-Elmer A100 flame atomic absorption spectrophotometer, using metal ion standard solutions for instrument calibration (Sigma, Milan, Italy).

The concentrations of Al were determined in helium mode by inductively coupled plasma mass spectrometry (ICP-MS) on an ICP-MS instrument (Agilent 7500ce, Tokyo, Japan), using the Babington nebuliser and a Scott-type spray chamber. A nickel sampler and skimmer with 1.0 and 0.4 mm cone orifices, respectively, were used. Treatment of data was performed with the Agilent ChemStation software. ICP-MS operating conditions for determination of Al are described elsewhere [23]. Before analysis samples were diluted with MilliQ water, so that the measured Al concentrations ranged between 1 and 100 µg L^−1^.

### Metallothionein Determination

Total MT concentration was determined following a silver-saturation assay, described in detail elsewhere [24], [25].

### Immunohistochemistry (IHC)

The half-brains used for immunohistochemical analyses were fixed by immersion in buffered formalin, washed in phosphate saline buffer (PBS) 0.01 M pH 7.4, processed for paraffin embedding, sectioned sagittally at a thickness of 5 µm and mounted on slides with permanent positive charge (poly-L-lysine coated slides).

MT I-II IHC. Sections were incubated in 3% H_2_O_2_ in PBS for 10 min. Non-specific binding sites were blocked by a 30 min incubation with serum-free protein block (Dako Cytomation, Milan, Italy). Sections used for MT I-II staining were pre-treated with citrate buffer pH 6, then incubated overnight at 4°C with a monoclonal anti-MT I-II antibody raised in mouse (DakoCytomation, Milan, Italy) diluted 1∶50. After washing, sections were reacted for 30 minutes with EnVision® kit, developed with 3,3′-diaminobenzidine (DakoCytomation, Milan, Italy) and counterstained with hematoxylin. The sections were then dehydrated, cover-slipped with balsam and observed.

Glial Fibrillary Acidic Protein (GFAP) immunohistochemistry. Sections for GFAP staining were pre-treated with 0.1% trypsin in Tris/HCl pH 7.2 at 37°C for 10 min, then incubated 30 min at room temperature with an anti-GFAP polyclonal antibody raised in rabbit (DakoCytomation, Milan, Italy) with a 1∶200 dilution. Further processing of the slides was the same described for MT I-II.

For the tyrosine hydroxylase (TH) immunohistochemistry sections were permeabilized with Triton X-100 2% in PBS 0.01 M, pH 7.2 for 15 min, blocked in 2% bovine serum albumin and Triton X-100 1% for one hour, incubated overnight in anti-TH (Santa Cruz Biotechnology N-19, Heidelberg, Germany) raised in rabbit diluted 1∶100, followed by anti-rabbit Alexa 594 (Molecular Probes, Eugene, Oregon, USA) secondary antibody, 1∶350, for 40 min at 37°C. The intensity of the staining was evaluated by grading – (faint labelling of cell bodies), +/− (detectable cell bodies and fibers), + (moderate labelling), ++ (intense labelling) and +++ (very intense) by two independent observers. MT I-II and GFAP sections were photographed with an Olympic BX51 photomicroscope. TH slides were photographed using the resident software of a Leica epifluorescence microscope. All images were acquired with the same parameters in TIFF format. The images were mounted unaltered, and lettered using Corel Draw 12.

The specificity of the antibodies has been verified by replacing the primary antibody with normal swine serum. Under these conditions there was no immunostaining.

### Behavioural Tests

The behavioural tests were run in an isolated room during five days, with a four-day pause between days 2 and 3. Mice were weighted every day and tested neurologically with special emphasis on: general condition, deambulation, posture, righting from the side, placing reaction of hindlimbs, geotaxic reaction, avoiding of borders and equilibrium.

Tests were chosen to explore different cognitive, sensory, motor and emotional domains; data were analyzed with mixed design ANOVA using Statistica software version 5 '97 edition (www.statsoft.com). The significant level was set at *p*<0.05.

#### Open-Field test

This test was performed on day 1 to evaluate locomotor and exploratory activities, and emotional reactivity to a novel environment [26]. Mice were individually moved to a plastic cage (55×33×20 cm), with opaque walls and black floor, and video-recorded for 3 min. The software SMART 2.5 (2 Biological Instrument, Varese, Italy) was used to quantify: *a)* the overall distance travelled (in cm), as an index of motor activity; *b)* the number of rearings with both forepaws on the lateral walls, as an index of exploratory activity; *c)* the time spent without moving, as an index of freezing; and *d)* the number of fecal boli and urine drops, as an index of autonomic activation.

#### Pole test

This test was performed to evaluate bradykinesia and motor performance [27], [28]. Mice were placed head upward on a vertical pole (1,5 cm diameter×50 cm height), the latency (in seconds) to climb down until the four paws were on the ground was recorded. The maximum time was 120 seconds. This test was repeated on days 1, 2 and 3, thus providing a measure for long-term contextual and procedural memory.

#### Predatory aggression test

The emotional reactivity to a different animal species was tested on day 1, by putting an earthworm *(Lombricus terrestris)* on the homecage floor. The latency (in seconds) to the first attack was recorded, then the test was stopped. The maximum time was 10 min. This test requires the sensory detection of the prey, the selection of the appropriate behavioral pattern of attack and its correct execution [26].

#### Habituation/Dishabituation smell test

From the second day onward, mice were tested for their ability to discriminate novel olfactory stimuli and their short-term memory [29]. Mice were moved to an open-field arena and video-recorded as described above. Each animal was left undisturbed for 5 min to habituate. A plastic well (20×7 mm) containing 10 µl of water was then fixed to the floor (10 cm from the short and 16 cm from the long wall) for 2 min, and then removed for 1 min. This procedure was repeated 3 times to provide habituation, seen as a lack of interest for the introduction of already known stimuli. Then a well containing 10 µl thymol (1∶1000 in liquid paraffin) was introduced for three times. When a new odour is presented, the mouse should be aware of it and explore it for a longer time. After the third thymol presentation, a well containing camphor (1∶1000 in liquid paraffin) was presented for three times. The area around the well (17×18 cm) was considered the target zone, in which the following measures were taken: latency to the first entry into the target zone (in seconds), distance travelled in cm, time spent in it (in seconds), resting time (in seconds), number of entries. Data were analyzed with a three-way mixed design ANOVA, for the factors Group (CuA *vs* CuD), odor (no odor, thymol, camphor), repetition (the first, second, third).

### Statistical Analysis

The statistical analyses referred to the behavioural tests have been described in the former paragraph. Analyses on metal and MT concentration were performed using T-test, GraphPad software and they are presented as mean as + SEM. Results were labeled as follow: * *p*<0.05, ** *p*<0.01, and *** *p*<0.001.

## Results

### Metal Concentration

Metal ion concentrations were measured in several organs to assess the effect of the CuD diet on the general metal balance. A widespread decrease of metal content was observed in each of the four brain regions tested and for each metal investigated (Figure 1B–F). In particular, CuD mice showed a significant reduction of Fe, Al, Zn, and Ca in the frontal areas of the brain, concurrent with a highly statistically significant decrease of Fe, Al, Zn and Ca in the cerebellum. Al was markedly decreased also in the occipital area (Figure 1D). Altogether, the frontal area and the cerebellum appeared to be the two regions most affected by the metal depletion.

**Figure 2:**
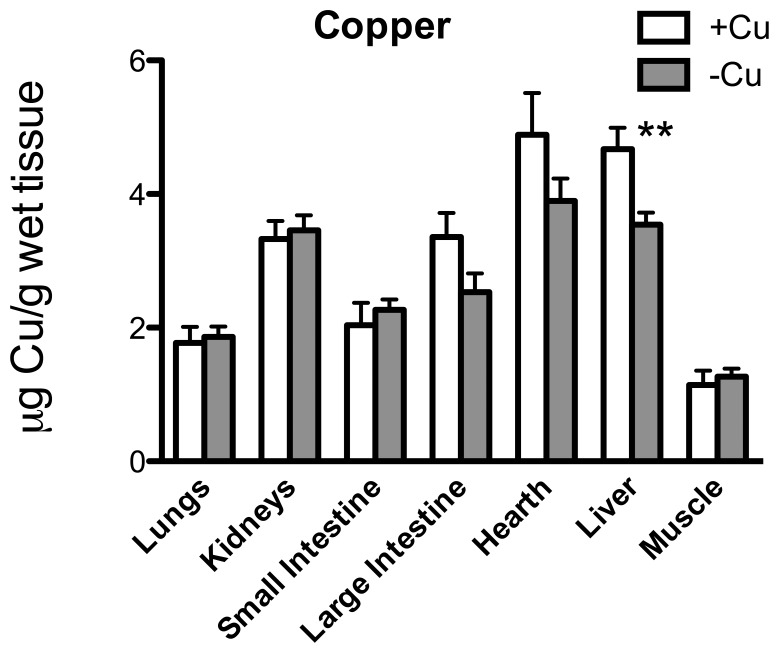
Cu content. Cu (µg/g wet tissue) in the lungs, kidneys, small intestine, large intestine, heart, liver, and muscle. ** *p*<0.01.

**Figure 3:**
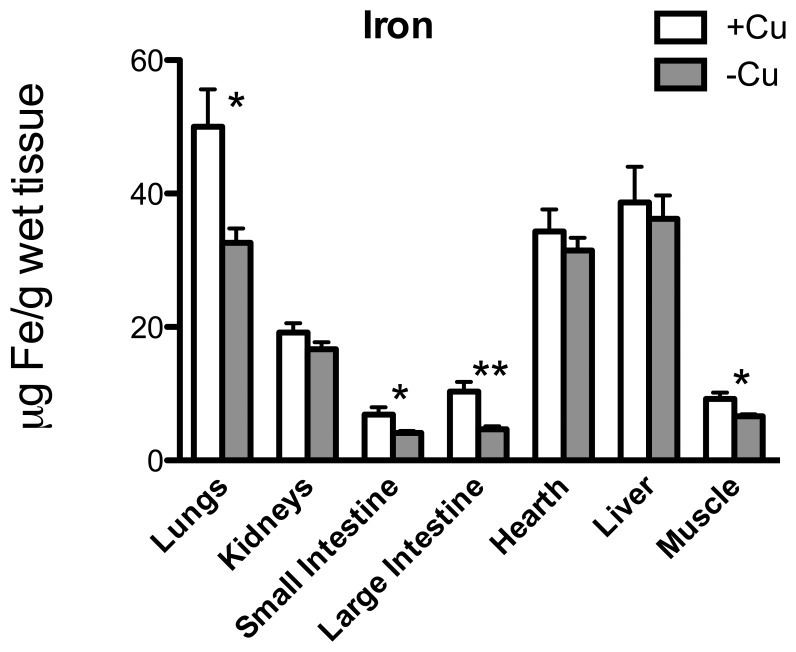
Fe content. Fe (µg/g wet tissue) in the lungs, kidneys, small intestine, large intestine, heart, liver, and muscle. * *p*<0.05; ** *p*<0.01.

**Figure 4:**
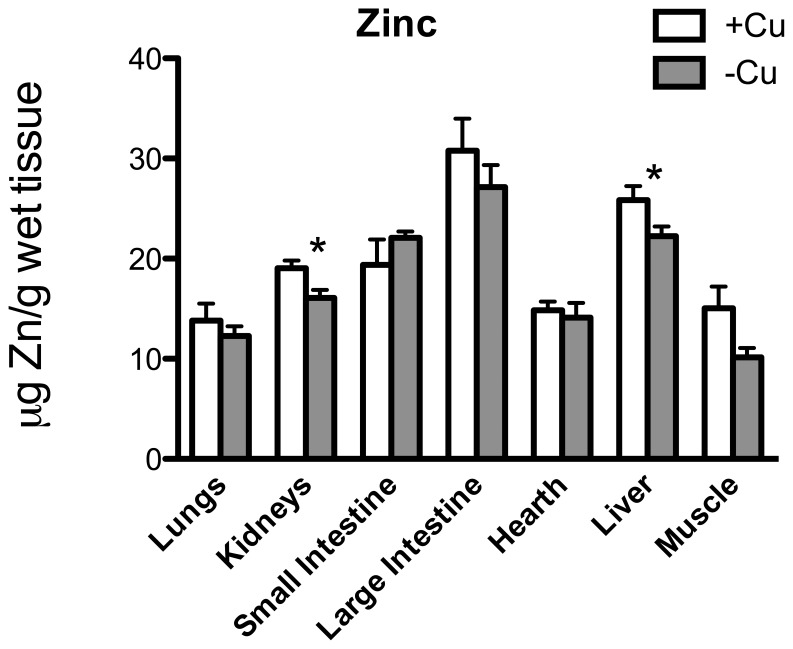
Zn content. Zn (µg/g wet tissue) in the lungs, kidneys, small intestine, large intestine, heart, liver, and muscle. * *p*<0.05.

**Figure 5:**
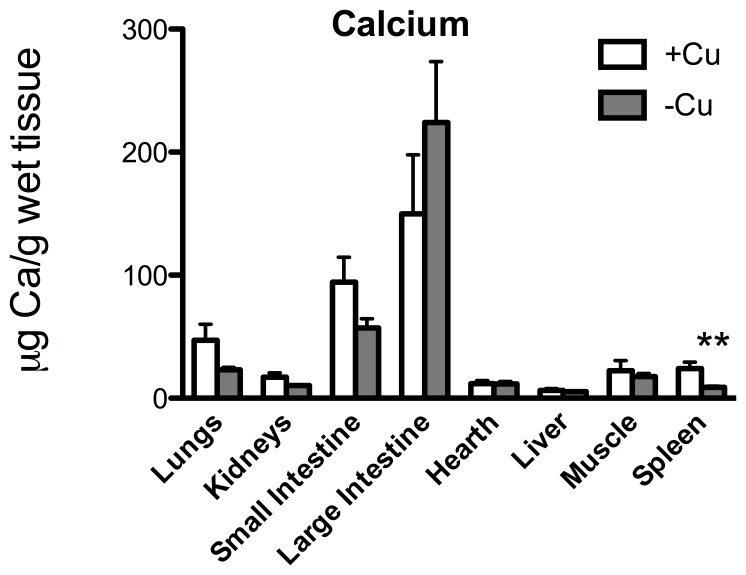
Ca content. Ca (µg/g wet tissue) in the lungs, kidneys, small intestine, large intestine, heart, liver, muscle, and spleen. ** *p*<0.01.

Figure 2 shows Cu concentration in the other main organs: liver was the only one showing a significant Cu decrement (*p* = 0.004). Fe was strongly curtailed in lungs (*p* = 0.012), small (*p* = 0.018) and large intestine (*p* = 0.0012) and muscle (*p* = 0.013) (Figure 3); Zn in kidneys (*p* = 0.013) and liver (*p* = 0.040) (Figure 4); and Ca in spleen (*p* = 0.004) (Figure 5).

### MT

**Figure 6:**
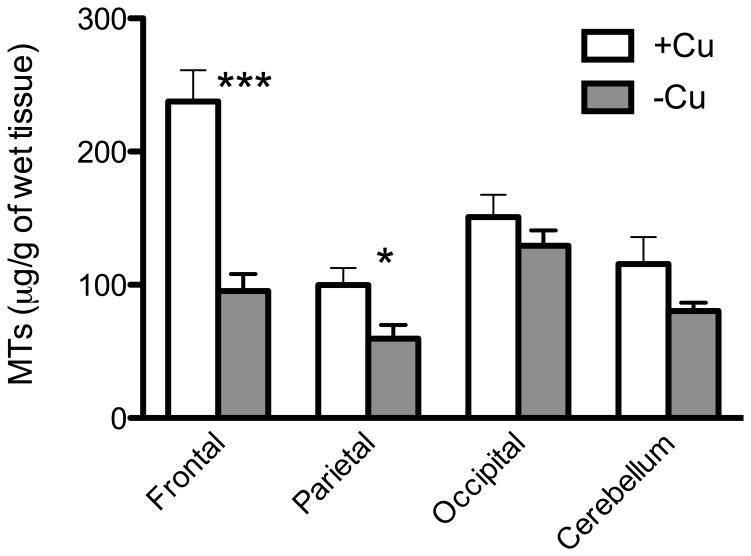
Metallothionein (MT) content in the brain. MT (µg/g wet tissue) in the parietal, frontal, occipital cortex, and cerebellum. *p<0.05; ***p<0.001.

The total amount of MT was significantly diminished only in the frontal (*p* = 0.0002) and parietal (*p* = 0.020) areas of the brain (Figure 6). Negligible was the difference in the MT concentration between CuA and CuD mice in the other organs (data not shown).

### Immunohistochemistry

Sections treated for immunohistochemistry were evaluated independently by three observers (see Table 1).

**Table 1:** Immunohistochemistry – Average of three independent evaluations.

Specimen	Tyrosine Hydroxylase	Metallothioneins I/II	GFAP
CTL 1	n.d.	+++	+++
CTL 2	n.d.	++	++
CTL 3	+/−	+++	++
CTL 4	n.d.	+	+++
CTL 5	++	++	++/+++
CTL 6	++	n.d.	++
CTL 7	++/+++	++	+++
CTL 8	n.d.	n.d.	n.d.
CTL 9	+	++	+++
CTL 10	++	+++	+++
CTL 11	+/++	++	+++
CTL 12	n.d.	n.d.	n.d.
EXP 1	++	+++	+++
EXP 2	++	+++	+++
EXP 3	+	++	+++
EXP 4	+++	++	+++
EXP 5	++/+++	++	++
EXP 6	+	+++	+++
EXP 7	+/−	++	++
EXP 8	++	++	++
EXP 9	n.d.	n.d.	n.d.
EXP 10	++	++	++
EXP 11	+/−	+	++
EXP 12	n.d.	n.d.	n.d.

CTL = control mice; EXP = copper deficient mice; n.d. not determined because of damaged specimen.

#### GFAP immunohistochemistry

GFAP-immunoreactive (-ir) elements were evident in both groups of mice without difference in distribution and density. Positive glial cells were mostly located in the white matter of the capsule system surrounding and crossing the basal ganglia complex, in the fimbria and hippocampus, in the pyramidal tract in the mesencephalon and in various fiber tracts of the medulla and in the white matter and cortex of the cerebellum. Positive elements were sometimes (but not always) found in the *locus cœruleus* of the CuD group but not in the controls (data not shown).

#### MT I-II immunohistochemistry

**Figure 7:**
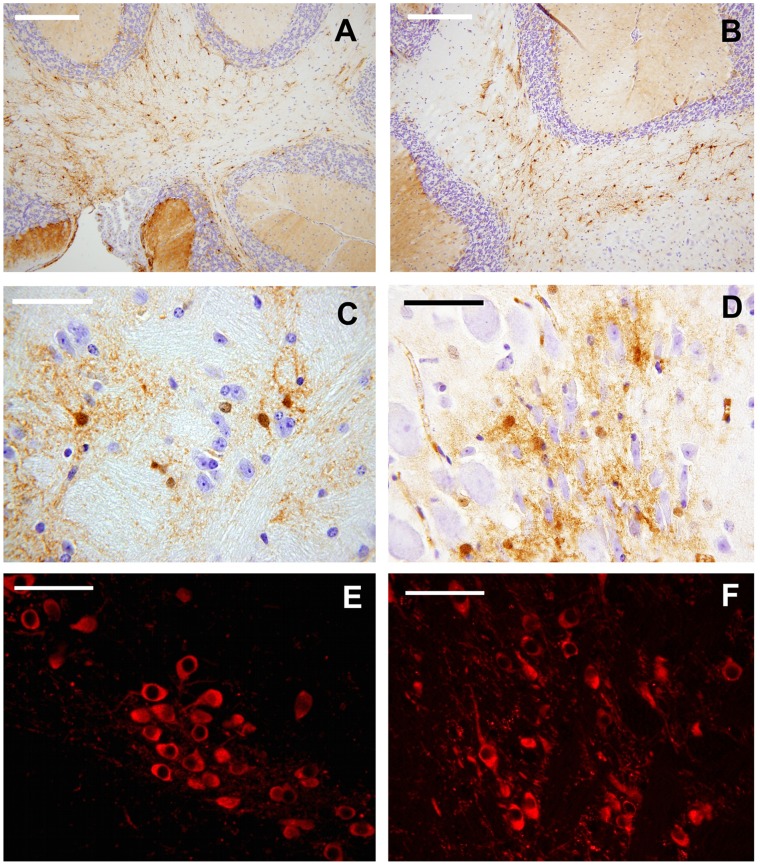
Representation of Immunohistochemical results. The images are relative to sagittal sections of the brain. Left column (A, C, E): CuD mice. Right column (B, D, F): control group. (A, B) MT I-II immunohistochemistry, cerebellum; scale bar = 200 µm; C, ventral tegmental area of the mesencephalon, scale bar = 50 µm; (D) *locus cœruleus*, scale bar = 50 µm; (E, F) TH-immunohistochemistry, (E) *substantia nigra*, scale bar = 50 µm.

MT I-II-ir cells were abundant and similarly localized in the CuD- and in the control group (Figure 7 A–D). MTI-II-ir elements were often associated to fiber tracts, including the capsule system of the forebrain, pyramidal tract and other tracts of the brainstem, and cerebellar peduncles (Figure 7 A_,_ B) and white matter. Positive elements were mostly evident in the caudate nucleus and putamen, hippocampus, fimbria, lateral nuclei of the septal area, central and ventral tegmental area of the mesencephalon, *locus cœruleus* (Figure 7 C_,_ D), motor nucleus of the facial nerve and other nuclei of the pons-medulla. Some (but not all) of the CuD animals showed a higher density of positive elements in the cochlear and vestibular nuclei. However, this latter result could be related to imperfect matching of sections, as differences of a few µm lateral to the sagittal plane may alter precise comparison of topography of smaller nuclear complexes.

#### TH immunohistochemistry

Representative images are shown in Figure 7 E, F. TH-ir neurons in the *substantia nigra*-ventral tegmental area appeared slightly more intense in the brains of mice receiving the CuD diet, showing more intense cell bodies and labelled fibers.

### Behavioural Tests

In the open field test, no difference was detected between controls and CuD mice in the distance travelled, F[1], [21] = 0.071, p = 0.792, in the number of rearings, F[1], [21] = 0.022, p = 0.883, in the resting time, F[1], [21] = 0.0237, p = 0.879, in the number of urine drops, F[1], [21] = 0.913, p = 0.350 and in the number of fecal boli, F[1], [21] = 1.434, 0 = 0.244.

**Figure 8:**
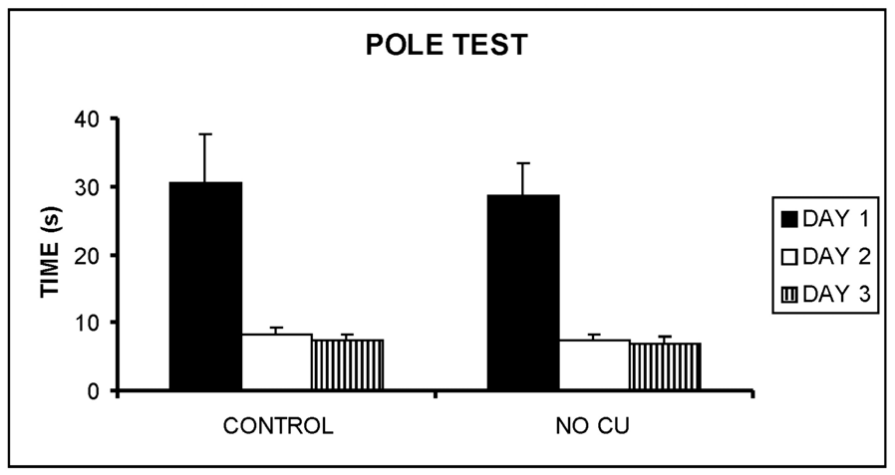
Pole test. Means+SEM are shown. Both groups can learn efficiently to climb down from the pole.

The pole test (Figure 8) suggested that no significant difference was present between the groups that behaved in the same way, F[1], [21] = 0.049, p = 0.826 the first day, F[1], [21] = 0.406, p = 0.531 the second day, F[1], [21] = 0.161, p = 0.692 the third day.

The predatory aggression test did not show any difference in the latency to attack the prey, F[1], [21] = 1.347, p = 0.258.

The habituation-dishabituation test showed no difference between the groups in each variable considered. Concerning the distance travelled in the target zone, the factors Odor and Repetition were significant, F[2], [42] = 9.489, p<0.0005 and F[2], [42] = 10.679, p<0.0005 respectively. These results are best explained by the interaction Odor×Repetition, F[4,84] = 2.772, p<0.05: when water was presented, a similar distance was travelled in each of the three presentations. When camphor was presented, both the second and the third presentation induced mice to move for a shorter distance (p<0.005 compared to the first presentation). The latency to the first entry into the target zone was similar between the groups, since only the factor Repetition is different, F[2], [42] = 7.564, p<0.005, with a shorter latency to enter the target zone at the first presentation, compared to the second (p<0.005) and third (p<0.01).

When considering the overall time spent in the target zone, that embrace the time spent moving and resting, only the factor Repetition was significant, F[2], [42] = 10.033, p<0.0005, with the first presentation inducing a longer permanence in the proximity of the new stimulus (p<0.002 and p<0.001 compared to the second and third presentation, respectively). A similar result was obtained for the resting time in the target zone; the factor Repetition resulted significant, F[2], [40] = 6.884, p<0.005, with the first presentation inducing a longer resting in the target zone, p<0.005 and p<0.01 compared to the second and third presentation.

When considering the number of entries in the target zone, the factor odor was significantly different, F[2], [42] = 10.143, p<0.0005, with the first condition (no odor) inducing a higher number of entries (p<0.005 and p<0.001 compared to thymol and camphor, respectively). The factor Repetition was different, F(2,42) = 25.660, p<0.05, with the first presentation inducing a higher number of entries, compared to the second and the third (p<0.05 for both).

## Discussion

Several studies have evaluated the effect of Cu deprivation and have concluded that this element is essential for brain growth and maturation [30], [31]. On the other hand, to the best of our knowledge, the investigations related to the effects of experimental diets on aged mice are scarce, notwithstanding its relevance for human nutrition strategies in old age. In addition, it is well established that a deficiency or excess in one metal can also influence the uptake of the others [32], [33], [34]. Our results confirmed this cross-interaction and, interestingly, demonstrated that also the concentration of Al was markedly altered in the brain of CuD animals compared to the controls.

Severe Cu deficiency can determine changes in Fe metabolism and can impair the correct absorption of dietary Fe [32]. Accordingly, we found decreased level of Fe in the brain (Figure 1C), particularly in the occipital area and in the cerebellum, as well as in the large and small intestine. It has been demonstrated that dietary Cu-deficiency induces anaemia that does not improve with Fe supplementation, however, it is fully reversed upon Cu supplementation [35].

In our experimental study we did observe neither liver nor intestinal Fe accumulation as described by other authors [32], [36]. The mechanism for Fe accumulation may be related to a decrease of Cu-enzymes such as ceruloplasmin and hephaestin that are required for Fe transport [37], [38]. Symptoms of anaemia and low plasma Fe level were recorded in weanling rats within days after treatment with a CuD diet [39]. One possible explanation for the discrepancy could be due to the different age of the animals. Pyatskowit and Prohaska [40] fed CuD diet to two cohorts of mice and rat pups: only the rat pups showed lower plasma and brain levels of Fe. Noteworthy, humans with Cu deficiency and anaemia may have normal plasma Fe concentrations [41]. In our series of aged mice, we observed no weight change between the two groups, and Cu was curtailed only in the frontal cortex.

Thus, Prohaska’s laboratory has widely demonstrated the effect of Cu-deficiency in mice and rats when this nutritional restriction is imposed throughout gestation as well as lactation: as a result, brain Cu concentration resulted dramatically affected by the diet [42]. Notably, if the diet began shortly after birth the effect in terms of Cu-brain alteration was strongly limited [43]. This could explain the fact that, also in our experimental model, Cu seemed to be the metal less affected by the diet. On the other hand, Ca concentration was greatly affected by the diet (Figures 1F; 5). Studies on intracellular Ca homeostasis affected by Cu deficiency are limited. Ca mobilization from intracellular stores has been reported to be influenced by Cu deficiency as Cu deficient platelets showed a diminished rinse in cytosolic Ca upon agonist administration [44]. Beside, Cu deficiency impaired the architecture of mitochondria [45], the activity of the permeability transition pore [46], and altered ATP synthase [47], [48] suggesting that Cu deficiency may exert effects in the electron transport chain and oxidative phosphorylation system causing Ca mobilization.

Independently of metals being a primary cause or a consequence of the disease mechanism, a change in a single metal ion will upset the whole elemental homeostatic pool, resulting in a significant imbalance in the elemental levels in the body (serum, CSF and brain) as a sort of domino effect. The effect of an increase or decrease of a single metal is not restricted to the initiating metal alone, and such changes affect the total elemental and charge distribution pattern in the nervous system.

The role played by MT in the detoxification of heavy metals and the distribution of essential metals is clearly established but in recent years there has grown a wider appreciation of MT functions. The extensive presence of MT within the CNS and their altered expression as a common feature of several neurodegenerative diseases support the hypothesis of an important neurological function played by these proteins. These ubiquitous low molecular weight cysteine-rich proteins are present in mammals with four isoforms. Our MT I-II IHC data indicated that the metal-bearing system did not seem to be modified in the brain of the mice fed with the CuD diet (Figure 7 A–D). In fact, the presence and the distribution of MT I-II were similar in the experimental series and in the control group. On the other hand, the total content of MT in the brain was dramatically affected by the diet (Figure 6). We speculate that the decrease of MT content in the frontal area could be due to the correlated decrease of Cu and Zn in that area. Many studies have shown the critical role of MT in the regulation of zinc homeostasis [49], [50], [51]. Particularly, under oxidative stress conditions, Zn can be released from MT to exert protection. Thus, if an oxidative reaction triggers Zn release from MT, a dynamic change in Zn concentration can occur [52]. Concerning trivalent metals, alteration in their content is not likely solely to be due to alteration of these proteins and other transporter proteins must be involved. The dramatic decrease of total MT content, especially in the brain, could be detrimental in the light of their function as free radical scavenger. It has been demonstrated that animals as well as cell culture deficient in MT isoforms exhibited greater susceptibility to oxidative stress caused by different stimula [53], [54]. The mechanism by which MT exert a protective function is not clear. However, a reaction between the MT thiolate cluster and hydroxyl radicals has to be envisaged [55].

Furthermore, our findings indicated that the CuD diet did not influence the distribution of the classical astrocyte supporting elements, these latter identified by GFAP immunohistochemistry. Also the TH system showed only minor modifications in the two groups of mice (Figure 7 E, F).

Mice fed either diet were similar in their general appearance and did not show any overt sign of distress. The open field test showed that CuD mice had no motor impairment and did not differ from controls in their exploratory activity, since both groups recognized the open field as a new environment and behaved consequently.

The pole test evaluates motor performance, but its repetitions in different days allowed also the assessment of long-term memory. Again, both groups of mice behaved similarly: the first day both groups took a long time to reach the ground, while the subsequent days both groups were equally fast in reaching the ground. This means that mice had no motor deficit, but also that both groups could learn, retaining the information for one or more days (four days between the second and third repetition of the test).

The predatory aggression test confirmed that no deficit was present in the sensory (mainly olfactory) perception of the prey, in the emotional reactivity to the prey, or in the motor execution of the attack sequence. This test refers to a complex sensory-motor and cognitive-emotional domain mainly mediated by basal forebrain structures and olfactory projection areas [26].

The habituation-dishabituation test was designed to evaluate the discrimination of novel olfactory stimuli and their retention in short-term memory [29]. The distance travelled in the target zone showed a progressive shortening in both groups, most probably due to the mice habituating to the test procedure.

The latency to the first entry into the target zone showed that both groups of mice entered equally fast in the area around the new stimulus, while both groups approached slower the stimulus, the second and third time it was presented. The overall time spent in the target zone confirmed that both groups of mice spent more time in the target zone the first time that a stimulus was presented. Therefore, both groups were able to discriminate the novel stimulus, which promptly elicited a fast exploratory behaviour, while the second and third presentation of the same stimulus induced a similarly slower response.

Although the array of alterations produced by the CuD diet in the aged mice appeared to be relevant, we did not observed any sign of impairments in their sensory, motor, emotional and cognitive behavioural patterns, compared to age-matched controls. The scenario is anyhow further compounded by the close interconnection among the different metals that can somehow create a sort of domino effect.

In conclusion, the fundamental biochemical mechanisms linking brain biometal metabolism, environmental metal exposure, and neurodegenerative pathophysiology appear to be rather complicated and warrant further investigation.
